# The Simple Mix Design Method and Confined Behavior Analysis for Recycled Aggregate Concrete

**DOI:** 10.3390/ma14133533

**Published:** 2021-06-24

**Authors:** Chong Rong, Jing Ma, Qingxuan Shi, Qiuwei Wang

**Affiliations:** 1 State Key Laboratory of Green Building, Western China of Xian University of Architecture & Technology, Xi’an 710055, China; shiqx@xauat.edu.cn (Q.S.); wqw0815@126.com (Q.W.); 2 College of Civil Engineering, Xi’an University of Architecture & Technology, Xi’an 710055, China; mktb1928@163.com; 3 Key Laboratory of Structural Engineering and Seismic Resistance Education, Xi’an University of Architectural & Technology, Xi’an 710055, China

**Keywords:** recycled aggregate concrete, RAC filled in steel tube, RAC filled in GFRP tube, mix design method, experimental study

## Abstract

For the environment protection and sustainable development in building construction, waste concrete can be processed into recycled aggregate to mix the recycled aggregate concrete (RAC). However, the existing mix design methods of RAC were complex, and the mechanical properties of RAC were more weakened than ordinary concrete. This paper presents a simple mix design method for RAC, including orthogonal test and single-factor test. Then, in order to study the behavior of confined RAC, this paper presents a comprehensive experimental study on the RAC filled in steel tube (RCFST) specimens and the RAC filled in GFRP tube (RCFST) specimens. The results show that the proposed mix design method can mix different stable strength grades of RAC promptly and efficiently. In addition, the steel tube and GFRP tube can provide a well confining effect on core RAC to improve the mechanical behavior of column. Moreover, the properties of core RAC in steel tube are the same as the common passive confined concrete, and the properties of core RAC in the GFRP tube are the same as the common active confined concrete. The study results can provide reference for other kinds of RAC mixtures as well as be a foundation for theoretical studies on confined RAC.

## 1. Introduction

To be an effective means of environmental protection, waste concrete can be made into recycled aggregate after crushing and screening. Then, the recycled aggregate is used to mix concrete as a replacement for natural aggregate, namely recycled aggregate concrete (RAC) [1,2]. Since the late 20th century, a growing number of studies have focused on the new environmental concrete [3,4]. Over the past two decades, many researches have studied some salient issues for the practical application of RAC: The production process and the compressive strength prediction are studied in the early stage [5,6,7]; the comprehensive mechanical properties and durability properties are studied over the past decades [8,9,10]; from the components and micro-properties of RAC to the macro-performance of RAC, the properties are studied in recent years [11,12,13,14,15,16,17]. Against the common concrete with natural aggregate, the RAC with a similar mix proportion has a weakened mechanical property and a great dispersion of properties, due to the weakened and uncertainty properties of recycled aggregate [18,19,20].

Some researchers adopt a series of methods to improve the weakened and dispersion properties of RAC. Evangelista [21] made waste concrete into fine recycled aggregate to mix RAC, but the replacement is low. Nam [22] studied the fiber reinforcement on the mechanical behavior of RAC, which showed that fibers and fine recycled aggregates played an important role. Gao [23] used the fine recycled aggregates, fibers, and high-activity binding materials to prepare high ductility cementitious composites. Meng [24] analyzed a relatively large database of RAC, then proposed a strength-based mix design method. However, the required strength ranged from 30 to 60 MPa in most engineering structures. In addition, the uncertainty properties of recycled aggregates and binding materials cause different mix proportions for the same target strength. Therefore, the methods to greatly improve the self-properties of RAC is complex and unnecessary. It is necessary to propose a mix design method which could be widely applied for the practical engineering of RAC to achieve the effect of quickness and simplicity.

In addition to the self-property improvement of RAC, the properties of RAC can be improved by the section form design in a composite structure. Based on the combinations of different materials, high property materials can be used to confine the concrete structure, while the confining materials can greatly improve the mechanical properties and durability properties of concrete [25,26,27]. The steel tube and FRP tube have been widely applied in engineering and researches [28,29]. There are some studies on the RAC filled in steel tube and FRP confined concrete [30,31,32], including the influence factors of triaxial compressive strength, the increase of peak strain, the prediction model for multi-axial strength, and the applications in RAC members. All studies show that the property of RAC can also be improved by the confining effect. However, the studies of the confining effect on stress-strain curves of core RAC in steel tube and FRP tube are limited. It is necessary to have a comprehensive study on the mechanical behavior of RAC under different types of confining effects.

Against the background, this paper is elaborated by focusing on two objects, the simple mix design method and confined behavior analysis. In the first part, based on the orthogonal test method and the single-factor test, the main factors are found and the strength prediction equation is proposed. The required mix proportion can be computed through the proposed equation. The design target is to design an efficient and accurate mix proportion of RAC with the given recycled aggregate in practical engineering. The second part presents an experimental study on the confined mechanical behavior of RAC, including the RAC filled in steel tube (RCFST) specimens and the RAC filled in GFRP tube (RCFST) specimens. The experimental study discusses the actively confining effect and passively confining effect on the behavior of RAC. The research results are important for the theoretical studies on all kinds of confined RAC, including the constitutive model of confined RAC, the section design methods, and the applications of composite RAC structures.

## 2. The Simple Mix Design Method of Recycled Aggregate Concrete

The simple mix design method, including the orthogonal test method and the single-factor test method, were combined to determine the mix proportion of RAC in this section. The former was firstly applied to find the main factors. Then, the latter was applied to propose the mix design equation by the main factors as variation. Finally, the detailed mix proportion of RAC with the target strength is computed by the mix design equation.

### 2.1. The Orthogonal Test

The orthogonal experimental design is an efficient, fast, and economical design method, which selects a part of the representative horizontal combination for the test. Based on tests of multi factors and multi levels, the significance of each factor is analyzed to determine the main factors. Based on the existing studies on RAC [30,31], three factors (water-binder ratio, binder-aggregate ratio, and fly ash) would be studied for the influence law and significant degree of the compressive strength in this section.

#### 2.1.1. The Basic Properties of Material Component

**Figure 1:**
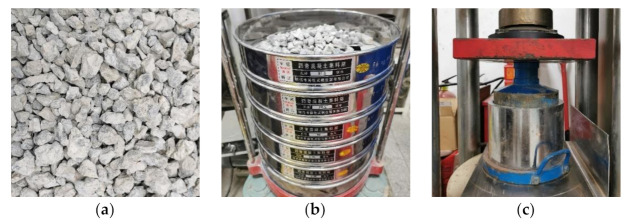
The recycled aggregate. (**a**) The processed recycled aggregate; (**b**) the size distribution test; (**c**) the crushing test.

The recycled aggregate was obtained from waste concrete members after crushing and screening processes, the processed recycled aggregate is shown in Figure 1a. The basic mechanical properties of recycled aggregate should be in accord with the Chinese Standard (GB/T 25177) [33]. The particle size distribution, crushing index, and apparent density were tested by the method in the Chinese Standard (GB/T 14685) [34]. In addition, the water absorbed rate was tested by the method in the Chinese Standard (GB/T 17431.2) [35]. The test processes are shown in Figure 1b,c.

In addition, three specimens were tested to ensure the accuracy of test results. Finally, the basic properties of recycled aggregate after the pretreatment are summarized in Table 1. Compared with the normal aggregate, the water absorbed rate and crushing index are bigger, which means that the recycled aggregate has weakened mechanical properties.

The particle size distribution is shown in Table 2. The fineness modulus of fine aggregate was 2.5, which belonged to the medium sand (the range is 2.3–3.0).

In the experiment, the cement used is ordinary Portland cement (P. O. 42.5), the fly ash used is Grade Ι, and the superplasticizer used is the polycarboxylate high performance superplasticizer of Subert. The water reducing rate is over 25 and the water used is ordinary tap water.

#### 2.1.2. Design of the Orthogonal Test

Based on the strength-based mix method, the 7- and 28-day compressive strength of RAC was taken as the test index. In addition, the water-binder ratio (A), the binder-aggregate ratio (B) which is the weight ratio, and the fly ash content (C) were taken as three variable factors. Each variable factor selected three levels in the test design: The water-binder ratio (A) included 0.30, 0.35, and 0.40; the binder-aggregate ratio (B) included 0.30, 0.33, and 0.36; and the fly ash content (C) included 10%, 15%, and 20%. The orthogonal design scheme *L*_9_(3^4^) was selected, the test needed nine specimens to study four variable factors with three levels. However, there were only three factors in our design, the fourth factor (D) was empty. Based on the Chinese Standard (JGJ 55) [36] and the orthogonal design scheme *L*_9_(3^4^), the test mix proportions design is shown in Table 3. In the experiment, the ratio of recycled aggregate was 100%, the moisture content of the aggregate was 0 due to the low water absorbed rate (see Table 1), and the dosage of superplasticizer was 1% of the binder weight.

**Figure 2:**
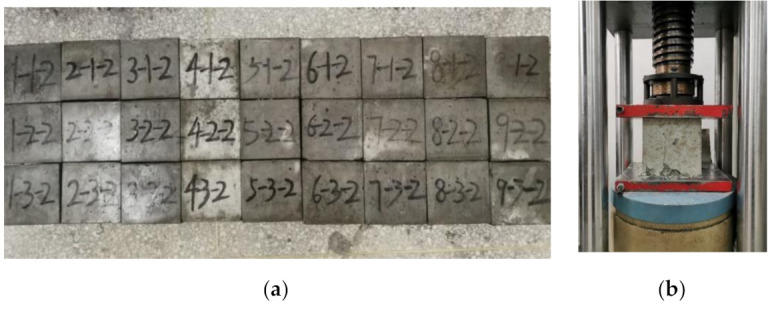
Compressive strength test of RAC cube specimens. (**a**) A part of the RAC cube specimens; (**b**) compressive strength test.

Nine concrete cube specimens (150 mm × 150 mm × 150 mm) of each mix proportion were poured, removed 24 h later and watered for curing at ambient temperature (Figure 2a). It should be noted that three of the specimens were tested after 7-days of curing and six were tested after 28-days of curing. The test was carried on the TYA-2000 electro-hydraulic pressure testing machine, as shown in Figure 2b.

### 2.2. The Orthogonal Test Results

Based on the compression test, the test results of 7- and 28-day compressive strength of RAC are summarized in Table 4. It should be noted that the compressive strength is the average value of test results of the same specimens. The test results in Table 4 illustrate that the strength increase trends of RAC with different mix proportions are similar. Furthermore, the range analysis and the variance analysis are used to discuss the test results in the following sections.

#### 2.2.1. Range Analysis

The range analysis is a common method to directly discuss the influence law of various factors. The range analysis results of 7- and 28-day compressive strength of the three factors are summarized in Table 5. In this table, *K*_i_ is the sum of the test values corresponding to one level in any factor column (i = 1, 2, 3), *k*_i_ is the corresponding average value, *R* is the difference between the maximum value and the minimum value of each level in any factor column. It is easy to understand that *R* is the parameter to evaluate these factors and the larger value illustrates greater influence of the factor.

Table 5 shows that *R*(A) > *R*(C) > *R*(B) for both the 7- and 28-day compressive strength. The sort order means that the water-binder ratio has the biggest influence, the fly ash content ranks the second, and the binder-aggregate ratio has the least influence. Meanwhile, the comparisons of three levels of each factor illustrate that the optimal values are the water-binder ratio (0.30), binder-aggregate ratio (0.30), and fly ash content (10%) for both the 7- and 28-day compressive strength.

**Figure 3:**
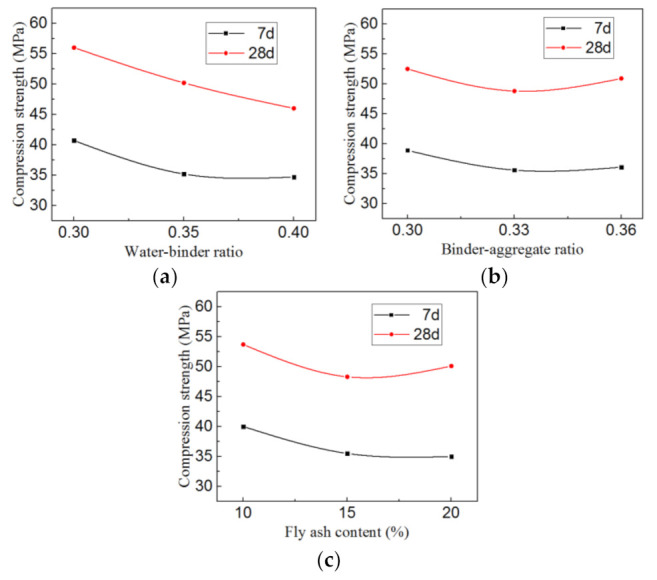
The relationships between the compressive strength and various levels of each factor (**a**) Water-binder ratio, (**b**) Binder-aggregate ratio, (**c**) Fly ash content.

In order to display the influence law of each factor visually and directly, the relationships between the compressive strength and various levels of each factor are shown in Figure 3a–c, where the level of each factor is abscissa and the *k*_i_ value of each factor column is ordinate.

In Figure 3, the following conclusions can be summarized: (a) The compressive strength decreases with the increase in the water-binder ratio, the relationship of 28-day compressive strength shows a line trend; (b) the compressive strength has an unclear change with various binder-aggregate ratios, which means that the binder-aggregate ratio has a slight effect; (c) the 7-day compressive strength decreases with the increase in the fly ash content, while the 28-day compressive strength with the fly ash (20%) has rallied slightly, probably due to the fact that the pozzolanic effect and micro aggregate effect of fly ash have not been fully stimulated at the early curing stage.

#### 2.2.2. Variance Analysis

There are two parts in the total variation of variance analysis, one part is the variation caused by factors, the other is the variation caused by errors. Based on the variance analysis (namely F examine), the significant degree of each factor on the compressive strength can be determined. Based on the comparison between the F value and critical value (F_a_), the significant degree can be divided into four situations: (1) F > F_0.01_ means particularly significant impact, marked as “**”; (2) F_0.01_ > F > F_0.05_ means significant impact, marked as “*”; (3) F_0.05_ > F > F_0.1_ means certain impact, marked as “(*)”; (4) F < F_0.1_ means little impact.

The variance analysis results of 28-day compressive strength are summarized in Table 6. The analysis results illustrate that the water-binder ratio has a significant impact on the 28-day compressive strength, the fly ash content has a certain impact on the strength, and the binder-aggregate ratio has a little impact on the strength. The analysis results are important for the development of the mix design method for RAC. The analysis results are also the foundation for the single-factor test in the following section.

### 2.3. The Single-Factor Test and the Mix Design Equation

The single-factor design method is a detailed and simple design method, which could be conducted in a further study after the orthogonal test. In the single-factor test, one main factor is set as the variable to analyze the effect of multi levels, while other factors are set as fixed values. Based on the test results, the fitting equation can be established.

Based on the orthogonal test results, the water-binder ratio is the main factor of compressive strength of the RAC. Therefore, the water-binder ratio was an independent variable in the single-factor test for the 28-day compressive strength, and the parameter values are 0.30, 0.34, 0.38, and 0.42. Meanwhile, the optimal binder-aggregate ratio was 0.30 and the optimal fly ash content was 10% of binding materials. Based on the above parameters, the mix proportion of specimens in the single-factor test are shown in Table 7. The experimental program of the specimens single-factor test is similar to that of the orthogonal test.

**Figure 4:**
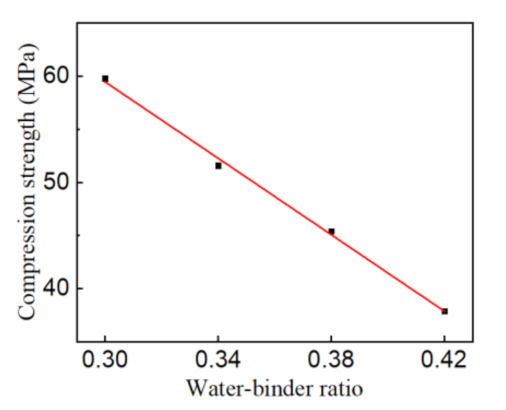
The relationship between the compressive strength and water-binder ratio.

The relationship between the 28-day compressive strength and water-binder ratio is summarized in Figure 4. It is easily found that the compressive strength decreases linearly with the increase in the water-binder ratio.

Based on the above linear relationship, the mix design method of RAC could be established as a linear equation, as shown in Equation (1). For the mix design formula, the binder-aggregate ratio is 0.30, the fly ash content is 10% of binding materials, which leads to the 28-day compressive strength of RAC as the target value, and the water-binder ratio is the variable parameter.

(1) $$f=113.4-179.8\left(W/B\right)$$

where *f* is the standard cube compressive strength of RAC and *W*/*B* is the water-binder ratio.

It should be noted that the proposed equation is only suitable for a given condition (i.e., recycled aggregate source, moisture content, absorption, and gradation) in this paper. The independent variables and coefficient values in the mix design equation must be changed in different conditions. A simple mixture design method was just suggested for RAC in different conditions. The design process of this method is described in the following section.

### 2.4. The Simple Mix Design Method

**Figure 5:**
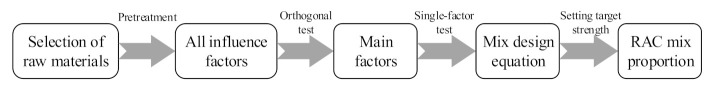
Process of simple mix design method of RAC.

In summary, the simple mix design method of RAC can be illustrated in a flow chart (see Figure 5). Furthermore, the flow chart consists of the following steps: (1) Based on the practical engineering, the raw materials are selected, including different recycled aggregates, cement type, binding material type, coarse aggregate type, grading of aggregate, fine to coarse aggregate ratio, concrete admixture, etc.; (2) all influence factors for strength are listed in the study; (3) based on the orthogonal test, the main factors are determined as variable, and secondary factors are set as fixed values; (4) based on the single-factor test, the mix design equation is established; (5) according to the target strength, the RAC mix proportion is computed by the mix design equation.

## 3. Experimental Program of Confined RAC Columns

Based on the existing researches [37,38], the mechanical properties of RAC are affected by the recycled aggregate, due to the weakened properties of recycled aggregate and interface layer between the cement paste and aggregate. It is generally believed that the lateral confining effect is provided by the high property material jacket, including steel tube and FRP [28,29,39]. According to the different confining mechanisms, three kinds of specimens would be studied in the following section: The unconfined RAC columns (RCC), RAC filled in steel tube (RCFST), and RAC filled in glass-FRP tube (RCFGT).

### 3.1. Specimens

In total, 17 specimens were prepared and tested in this experiment, including one triplet of RCC (three specimens), three pairs of RCFSTs (six specimens), and four pairs of RCFGTs (eight specimens). The design sizes of all test specimens were summarized in Table 8. For convenience of analysis, each test specimen was given a name: The name starts with several letters to distinguish the specimen type, followed by a number (i.e., 1, 2, 3 or 4) which indicates the size of the steel tube or GFRP tube, the name ends with a number after a dash (i.e., 1, 2 or 3) to distinguish the two or three identical specimens of each pair. It is noted that the sizes of RCFSTs are different from other specimens, due to the fact that these specimens are used to analyze more complex section forms in the following future research, including multiple confined RAC and complex confined RAC. Therefore, the stress-strain curve trends and the stress-strain data were greatly studied in this paper. The former was used to study the behavior of core RAC in the steel tube. The latter was acknowledged as original data for theoretical analysis.

**Figure 6:**
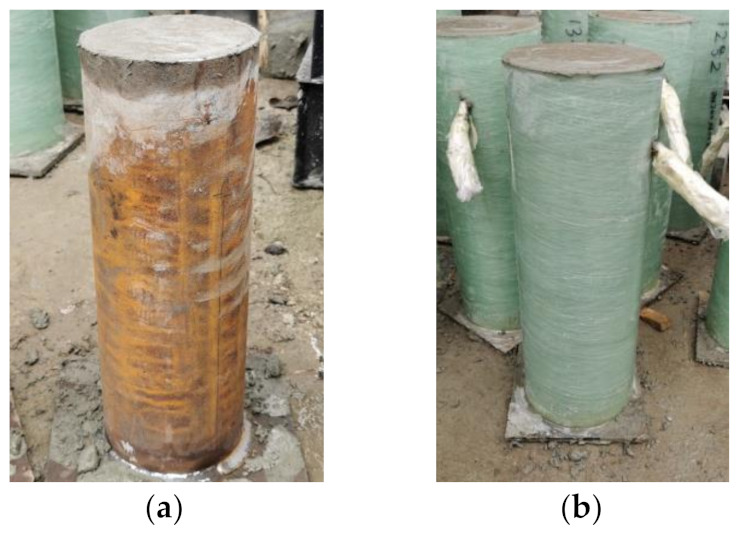
The confined RAC specimens. (**a**) RCFST; (**b**) RCFGT.

In preparation of the specimens, the steel tubes and GFRP tubes were prefabricated in the factory and used as formwork, the bottom of the steel tubes was sealed by a welding steel plate and the bottom of the GFRP tubes was sealed with silicone gel and board to avoid water leakage. Based on the mix design method in Section 2, the RAC was mixed and poured in steel tubes and GFRP tubes in several batches. Three RAC test blocks were poured in each batch to minor the concrete strength of different batches. All of the specimens and test blocks were cured at ambient temperature over 28 days, as shown in Figure 6. In addition, for the unconfined RAC columns, GFRP tubes were removed after the curing progress over 7 days.

To study the behavior of GFRP confined RAC columns under the weakened confining effect, the GFRP tube should be thin enough. However, only the GFRP tubes with 5- and 8-mm thickness were available in the market, which provided a large confining effect. Therefore, the specimens of RCFGT1 and RCFGT2 were prepared by wet-wrapping the GFRP cloth on the unconfined RAC column after 28-days of curing. The thickness of the wrapped GFRP in each specimen was shown in Table 6. The lap length of the wrapped GFRP was 150 mm. It is noted that the wet-wrapping of GFRP is different from the GFRP tube, while the former does not have axial bearing capacity.

### 3.2. Material Properties

**Figure 7:**
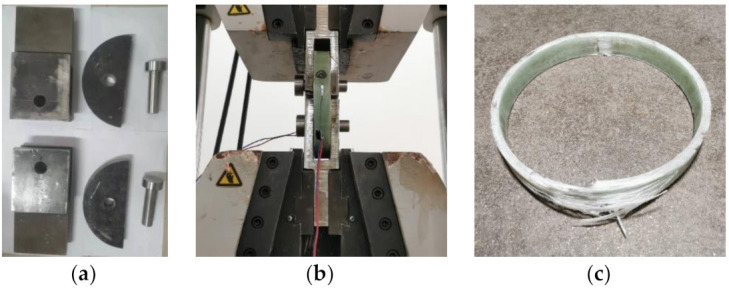
The material property test of GFRP tube. (**a**) The separation plate; (**b**) test process; (**c**) the GFRP coupon after the test.

For RCFGT3 and RCFGT4 specimens, the GFRP tubes were prefabricated in a factory, the winding angle of GFRP tubes was 80°, and the thicknesses were 5 and 8 mm. According to Reference [40], the tensile test of the GFRP tubes were conducted using the separation plate (Figure 7a). The test process was conducted in the following steps: (1) The GFRP tube was cut into a 30 mm-wide ring; (2) the ring was installed on the outer edge of the separation plate; (3) two circumferential strain gauges were pasted on both sides of the GFRP ring surface close to the separation plate gap; (4) the assembled coupons were tested using the WAW-1000 microcomputer controlled electro-hydraulic servo universal testing machine (Figure 7b), while the GFRP coupon after the test was shown in Figure 7c. The coupon test results are listed in Table 9.

For RCFGT1 and RCFGT2 specimens, the GFRP tube was directly fabricated by a wet-winding process on RAC columns, the thicknesses were 1 and 2 mm. Three GFRP coupons of each thickness GFRP tube were fabricated, and the tensile test was conducted on these GFRP coupons according to Reference [41].The average results of coupon test are listed in Table 9.

For the three types of steel tubes in RCFSTs, three steel coupons were cut from each size of steel tube in the longitudinal direction, and tensile tests were conducted on the steel coupons according to Reference [42]. The average results of the coupon test are listed in Table 10.

**Figure 8:**
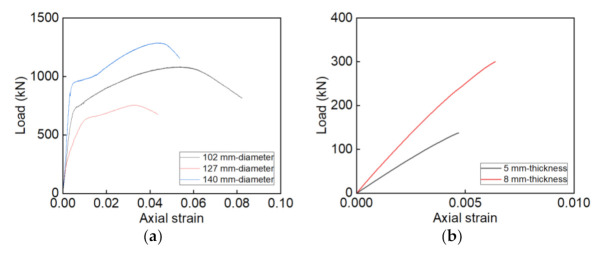
The load-axial strain curves. (**a**) Steel tubes; (**b**) GFRP tubes.

To reflect the axial compressive behavior of confining material, the load-axial strain curves of steel tubes and GFRP tubes are shown in Figure 8. The axial strain of these curves is the ratio of displacement measured by two LVDTs to the column height. It is apparent in Figure 8 that the curves of steel tubes include the elastic stage, harden stage, and soften stage, which correspond to the elastic state, yield state, and local buckling state of steel tubes. The curves of GFRP tubes show linear trends. These observations are important for the experimental analysis of the stress-strain curve of core RAC in the steel tube or GFRP tube.

### 3.3. Test Setup and Instrumentation

All of the specimens were tested under concentric compression. The column ends of each specimen were in direct contact with the loading plates of the test machine. It is noted that the loading plate is able to directly load the tube. The column ends should be leveled by the high strength gypsum before the test. For each specimen, three axial strain gauges and three lateral strain gauges were attached on the column surface at mid-height. In addition, the three same strain gauges belonged to the equidistance distribution along the circumferential direction. Two linear variable displacement transducers (LVDTs) were diagonally placed between the two loading plates of the test machine and used to measure the shortening of total column.

**Figure 9:**
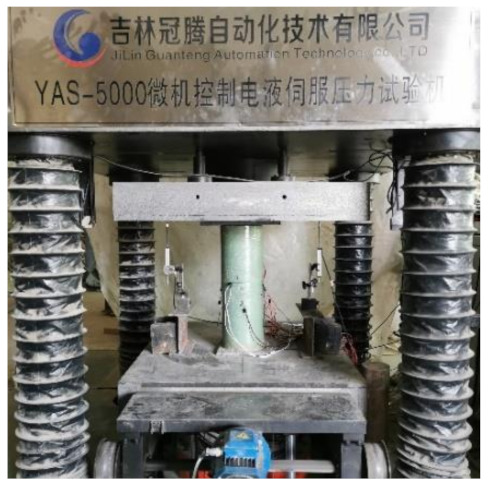
Specimen in test.

The test was conducted by the displacement controlled loading method on the YAS-5000 microcomputer controlled electro-hydraulic servo pressure testing machine. The loading rate of RCC, RCFGT1, and RCFGT2 was 0.003 mm/s. The loading rate of RCFGT3 and RCFGT4 was 0.007 mm/s. The loading rate of RCFSTs was 0.005 mm/s before steel tube yielding and 0.012 mm/s after steel tube yielding. The photo of the specimen in the test is shown in Figure 9.

## 4. Test Results and Discussion

### 4.1. Test Observation and Failure Modes

**Figure 10:**
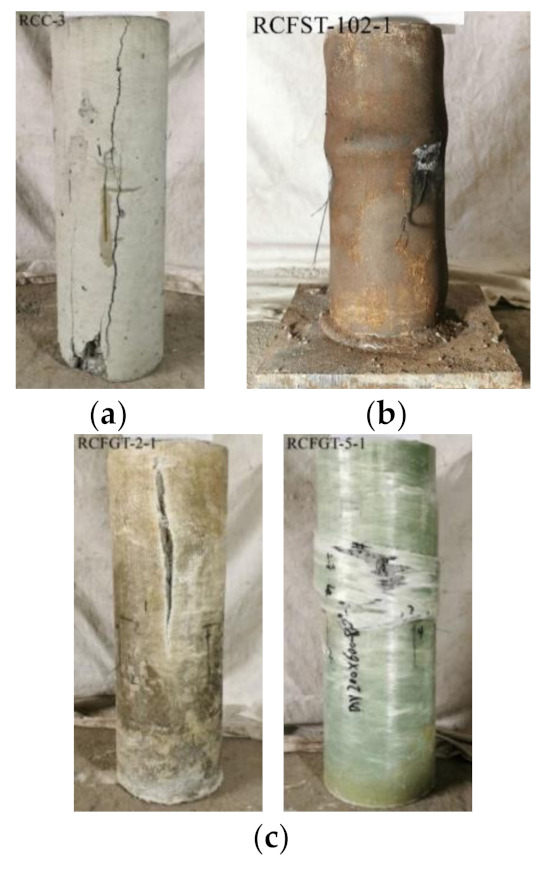
The failure of specimens. (**a**) RCCs; (**b**) RCFSTs; (**c**) RCFGTs.

For RCC specimens, the tests were terminated when vertical or oblique cracks occurred on the column surface and increased rapidly. The unconfined RAC columns failed suddenly, due to the loss of bearing capacity with vertical-through crack and partial crushing of RAC. The typical failure modes of RCC specimens are shown in Figure 10a. For RCFST specimens, the column surface had no obvious change in the elastic stage; the middle and the end of the steel tube showed external bubbling after the limit stage; then the local buckling of the steel tube still developed without the loading loss. The typical failure modes of RCFST specimens are shown in Figure 10b. For RCFGT specimens, the tests were terminated when the rupture of GFRP tube occurred. However, there were different failure modes of RAC in the GFRP tube: For the RCFGT specimens with a weakened confining effect, the failure part was largely distributed along the vertical direction and the crack occurred and developed on the core RAC column; for the RCFGT specimens with a large confining effect, the failure part was focused on the mid-height of the column and the core RAC crushed in the failure sections. The typical failure modes of RCFGT specimens are shown in Figure 10c.

### 4.2. The Load-Displacement Behavior

**Figure 11:**
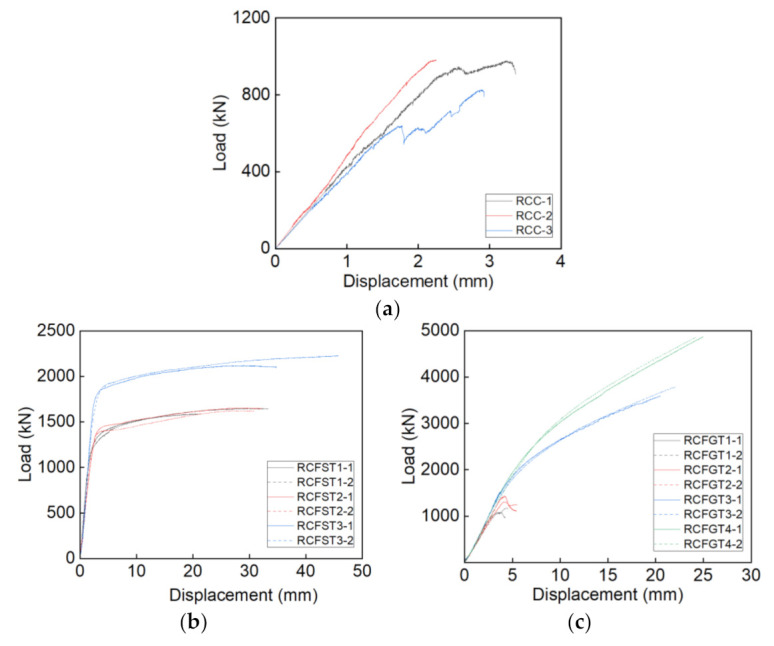
The load-displacement curves. (**a**) RCC; (**b**) RCFST; (**c**) RCFGT.

Figure 11a shows the load-displacement curves of the RCC specimens, the descending sections of curves are not measured, due to the brittle failure of RAC. Based on the test results, the following curve characteristics can be drawn: (1) The elastic stage is a large part in the ascending stages; (2) the specimens fail suddenly after the peak load; (3) the load increases slowly after suddenly falling down. This is due to the fact that: The high brittleness leads to the large elastic stage of RAC; the weak properties lead to the early crushing of recycled aggregate and some crushing aggregate would bear the loading again, due to the filling effect.

The load-displacement curves of the RCFST specimens in Figure 11b show that: The steel tube and core RAC work well together in the elastic stage; the load remained stable later in the loading stage; and the specimens had high ductility. The test results show that the steel tube can well confine the core RAC column.

Figure 11c shows that the GFRP tube has an obvious effect on the mechanical behaviors of RCFGT specimens, due to the confining effect on the core RAC. For the specimens under the low confining effect, the load drops after the peak point, and then tends to rise gently, such as RCFGT1 and RCFGT2. For the specimens under the high confining effect, the load-displacement curves show bilinear trends, such as RCFGT3 and RCFGT4. It should be noted that the curve of RCFGT2-1 is close to the horizontal growth after the peak point. The trend shows that the confining effect of RCFGT2 is close to the boundary value of the two different trend curves.

### 4.3. The Load-Axial Strain Curves

**Figure 12:**
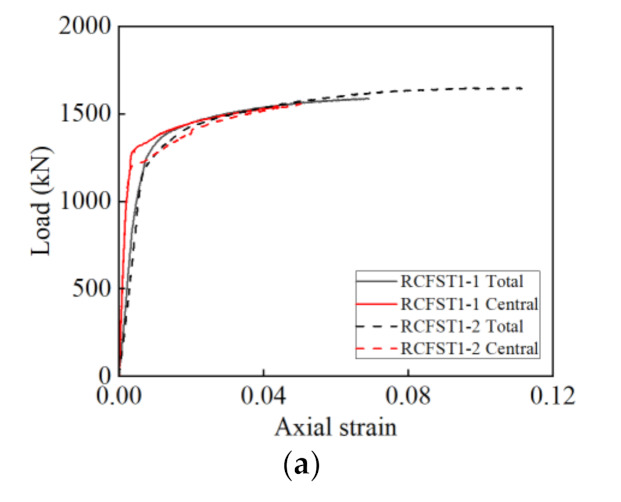

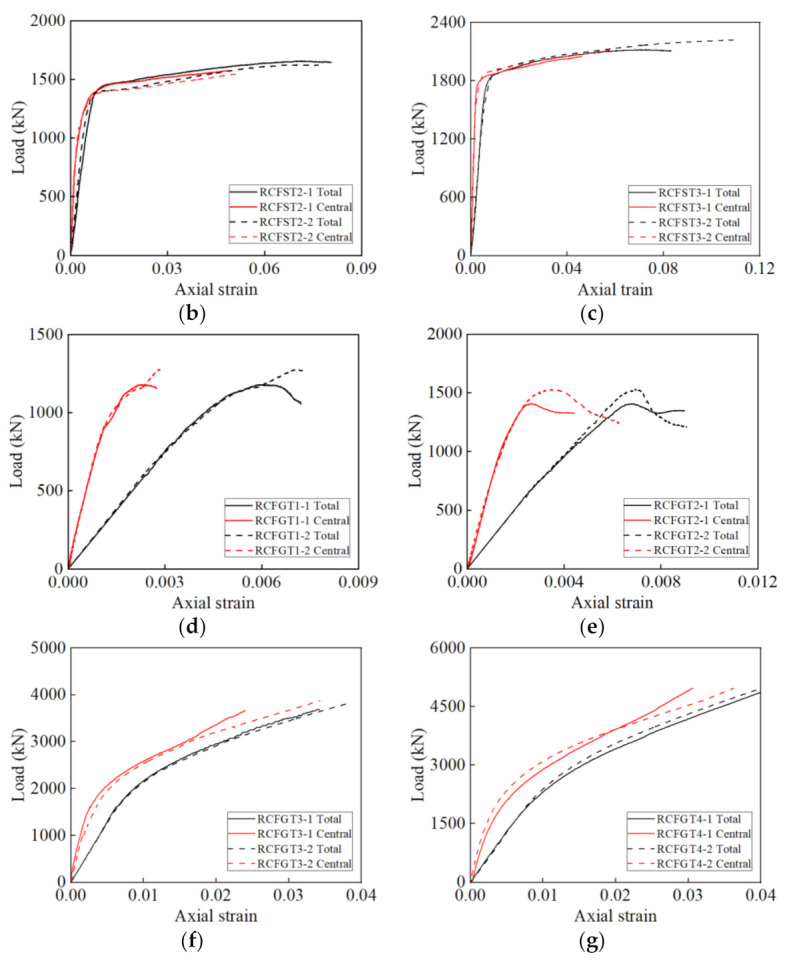
The load-axial strain curves. (**a**) RCFST1; (**b**) RCFST2; (**c**) RCFST3; (**d**) RCFGT1; (**e**) RCFGT2; (**f**) RCFGT3; (**g**) RCFGT4.

The comparisons in Figure 12 show differences between the axial total strain and axial central strain in the load-axial strain curves of the RCFST and RCFGT specimens. For the curves in Figure 12, the total strain is the ratio of displacement measured by two LVDTs to the specimen height, the central strain was measured by three axial strain gauges. The curves in Figure 12 show that: The growth of the central strain is much slower than that of the total strain in the first increasing stage, the central strain is less than 1/3 of the time of total strain in the early loading; the growth rate of the central strain increases to the similar rate of total strain at the following loading stage, the central strain is still less than the total strain of all specimens; and the central strain cannot be measured before the loading stop, due to the concrete damage or local buckling of steel tube.

In general, there is much difference between the central strain and the total strain, mainly due to the uneven expansion of mid-height concrete. The axial central strain cannot reflect the accurately stress state and deformation of the specimens. Therefore, the axial total strain is used in the behavior analysis in the following sections.

### 4.4. The Stress-Strain Curves of Core Recycled Aggregate Concrete

**Figure 13:**
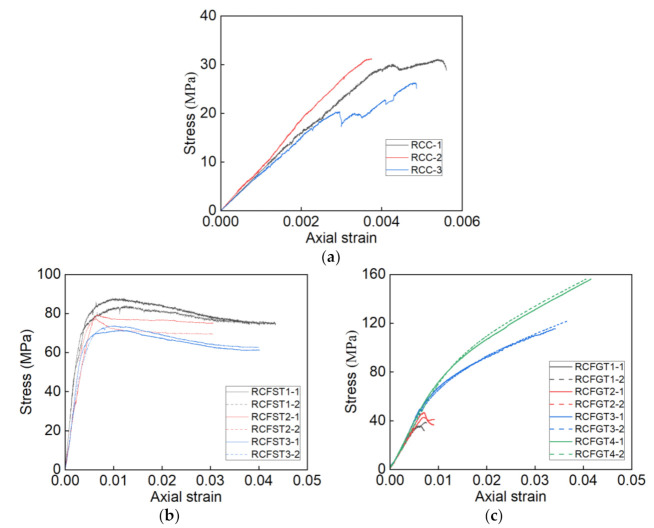
The stress-strain curves. (**a**) RAC; (**b**) core RAC in steel tubes; (**c**) core RAC in GFRP tubes.

As known in Figure 8, the steel tubes and GFRP tubes have obvious bearing capacities. The load provided by the steel tube or GFRP tube should be removed to compute the stress-strain curves of core RAC in the specimens [26,39]. The process is as follows: (1) Based on Figure 8 and Figure 12, getting the load points of core RAC which correspond to the axial strains is achieved by the load subtraction; (2) the computation load is then divided by the section area of core RAC to get the stress; (3) by a combination between each stress point and strain point, the stress-strain curve of core RAC is drawn up. The stress-strain curves of RAC, core RAC in steel tubes, and GFRP tubes are shown in Figure 13.

Figure 13a shows the stress-strain curves of unconfined RAC, the peak stress is around 30 MPa, the peak strain is around 0.004. The comparisons of curves in Figure 13 show that both the stress and strain have been greatly improved by the confining effect of the steel tube and GFRP tube.

Figure 13b shows that the RCFST specimens have high strength and ductility. It illustrates that the steel tube provides the well confining effect on the core RAC to improve the axial mechanical properties of specimens. All curves of core RAC of the RCFST specimens show similar two-stage trends, including an ascending stage and a gently descending stage. Meanwhile, the thicker steel tube provides the higher confining effect, which leads to the higher strength, well ductility, and smaller slope of descending stage. In addition, the typical behavior of steel confinement is that the lateral confining pressure is constant after steel yielding [39]. Therefore, the compressive behavior of the core RAC in the steel tube is the same as that of the actively confined concrete.

The comparisons between Figure 13c and Figure 12d–g show that the stress-strain curves of core RAC in GFRP are similar to the load-strain curves of RCFGT specimens, these kinds of curves have similar trends. The stress-strain curves of core RAC with the weak GFRP confining effect included the elastic stage, harden stage, and soften stage. The stress-strain curves of core RAC with the high GFRP confining effect show bilinear trends. In addition, the typical behavior of steel confinement is that the lateral confining pressure is always increasing, due to the no yielding of GFRP. Although the GFRP tube fails in a brittle manner, GFRP wrapping has some slower failure due to the progressive failure of fibers and triaxial stress state in the fibers [43]. Therefore, the compressive behaviors of the core RAC in the GFRP tube are the same as those of the passively confined concrete.

### 4.5. The Axial Strain-Lateral Strain Curves

**Figure 14:**
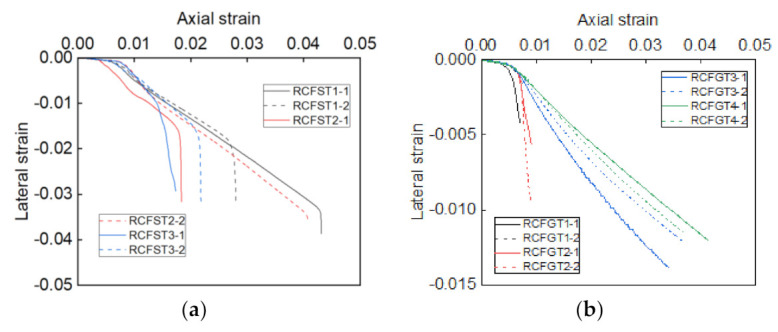
The axial strain-lateral strain curves. (**a**) Core RAC in steel tubes; (**b**) core RAC in GFRP tubes.

The axial strain-lateral strain curves of core RAC in steel tubes and GFRP tubes are summarized in Figure 14. Most of the curves show bilinear trends. The dilation ratio of the lateral is slow at first and then faster, and the later dilation ratio is almost kept at a constant state. The observation reflects that the expansion of core RAC is linearly related to the axial displacement in the later loading stage, where the axial strain is around 0.005. However, there are some differences in the dilation ratio of these specimens: The ratios of RCFST specimens are around −0.897, the ratios of RCFGT1 and RCFGT2 specimens are around −2.35, and the ratios of RCFGT3 and RCFGT4 specimens are around −0.3164. It means that the high confining effect would limit the expansion of core RAC.

## 5. Conclusions

This paper provided insights into the mix design method of RAC and the compressive behavior of confined RAC column through a detailed experimental study. The mix experiment included the orthogonal test method and the single-factor test method. The combination of the two methods improved the efficiency and accuracy of the mix design method of RAC. The compression experimental study included RAC filled in steel tubes and RAC filled in GFRP tubes. In addition, the sizes of RCFST specimens were different from that of the others used in the experimental study. The effects of confining types on various aspects of the core RAC behavior have been clarified in the paper. In summary, the following conclusions can be drawn:

(1) The strength-based mix design method for RAC is proposed. The proposed method suggests an effective and simple mixture way for the wide application of RAC in the practical engineering structure.
(2) The RAC columns under compression fail by the quick development of the vertical cracks on the column surface. The RAC shows an obvious brittle failure.
(3) Both the steel tube and GFRP tube have an obvious confining effect on the core RAC. In addition, the mechanical behaviors of RAC are greatly improved under the high confining effect, the strength was improved over two times and the failure strain was improved over ten times.
(4) The load development of the RCFST specimens under compression tends to be gentle, and the local buckling on the steel tube develops in the later loading stage. The stress-strain curves of core RAC in steel tubes show the characters of actively confined concrete, the ultimate stress is over 85% of the peak stress.
(5) The RCFGT specimens under compression fail by rupture of the GFRP tubes at the mid-height of the column. It can be noted that the failure stress was improved over four times and the failure strain was improved over nine times in RCFGT3 and RCFGT4. Meanwhile, the stress-strain curves of core RAC in GFRP tubes show the characters of passively confined concrete.

## Figures and Tables

**Table 1:** The basic properties of recycled aggregate.

Apparent Density (kg/m^3^)	Water Absorbed Rate (%)	Crushing Index (%)
2690	4.6	12

**Table 2:** The particle size distribution of coarse aggregate and fine aggregate.

Item		Sieve Diameter (mm)					
Coarse aggregate		<2.36	2.36	4.75	16.0	26.5	31.5
	Sieve residue (g)	16	7	1990	2958	27	0
	Each residue rate (%)	0.3	0.1	39.8	59.2	0.5	0.0
	Accumulated residue rate (%)	100	100	100	60	1	0
	Parameter in standard (%)	-	95~100	90~100	30~70	0~5	0
Fine aggregate		<0.15	0.15	0.30	0.60	1.18	2.36
	Sieve residue (g)	24	83	150	132	69	43
	Each residue rate (%)	4.7	16.5	29.9	26.4	13.7	8.5
	Accumulated residue rate (%)	99.6	95	79	49	22	9
	Parameter in standard (%)	-	90~100	70~92	41~70	10~50	0~25

**Table 3:** The mix proportion design of orthogonal test *L*_9_(3^4^).

Test Number	Water-Binder Ratio (A)	Binder-Aggregate Ratio (B)	Fly Ash Content (C)	Blank Factor (D)
1	0.30	0.30	10%	-
2	0.30	0.33	15%	-
3	0.30	0.36	20%	-
4	0.35	0.30	15%	-
5	0.35	0.33	20%	-
6	0.35	0.36	10%	-
7	0.40	0.30	20%	-
8	0.40	0.33	10%	-
9	0.40	0.36	15%	-

**Table 4:** The average compressive strength (MPa).

Item	1	2	3	4	5	6	7	8	9
7-day	46.3	37.7	38.1	35.8	32.5	37.2	34.5	36.6	33.0
28-day	59.9	52.4	55.7	49.7	46.8	54.1	47.9	47.2	42.8

**Table 5:** The range analysis results of 7-day and 28-day compressive strength (MPa).

Item		Influence Factor		
		A	B	C
7-day compressive strength	*K*_1_	122.1	116.6	120.1
	*K*_2_	105.5	106.8	106.5
	*K*_3_	104.1	108.3	105.1
	*k*_1_	40.7	38.9	40.0
	*k*_2_	35.2	35.6	35.5
	*k*_3_	34.7	36.1	35.0
	*R*	6.0	3.3	5.0
28-day compressive strength	*K*_1_	168.0	157.5	161.2
	*K*_2_	150.6	146.4	144.9
	*K*_3_	137.9	152.6	150.4
	*k*_1_	56.0	52.5	53.7
	*k*_2_	50.2	48.8	48.3
	*k*_3_	46.0	50.9	50.1
	*R*	10.0	3.7	5.4

**Table 6:** The variance analysis of 28-day compressive strength.

Item	Quadratic Sum	Free Degree	Mean Square Difference	F	Critical Value F_a_	Significant Degree
A	152.1	2	76.07	36.83	F_0.01_(2,2) = 99	*
B	20.6	2	10.32	4.99	F_0.05_(2,2) = 19	
C	45.8	2	22.90	11.08	F_0.1_(2,2) = 9	(*)
Error	4.1	2	2.07			
Total	222.6	8				

**Table 7:** The consumption of each mix proportion in the single-factor test (kg/m^3^).

Water-Binder Ratio	Cement	Fly Ash	Fine Aggregate	Coarse Aggregate	Water
0.30	446	50	562	1090	149
0.34	442	49	556	1080	167
0.38	438	49	551	1070	185
0.42	434	48	546	1060	202

**Table 8:** The design sizes of all test specimens.

Specimen	Column Diameter (mm)	Column Height (mm)	Confining Material Jacket		
			Type	Diameter (mm)	Thickness (mm)
RCC-1,2,3	200	600	None	-	-
RCFST1-1,2	102	300	Steel tube	115	6.5
RCFST2-1,2	127	400	Steel tube	138	5.5
RCFST3-1,2	140	400	Steel tube	153	6.5
RCFGT1-1,2	200	600	GFRP	202	1
RCFGT2-1,2	200	600	GFRP	204	2
RCFGT3-1,2	200	600	GFRP	210	5
RCFGT4-1,2	200	600	GFRP	216	8

**Table 9:** Material properties of GFRP tubes.

Thickness (mm)	Tensile Strength (MPa)	Elastic Modulus (MPa)	Fracture Strain
1	181	11,786	1.24%
2	190	13,972	1.13%
5	465	42,608	1.38%
8	473	53,062	1.29%

**Table 10:** Material properties of steel tubes.

Size (mm)	Yield Strength (MPa)	Ultimate Strength (MPa)	Elastic Modulus (MPa)
102 × 6.5	405	548	2.23 × 10^5^
127 × 5.5	340	507	2.10 × 10^5^
140 × 6.5	380	520	2.11 × 10^5^

## Data Availability

Data are available in a publicly accessible repository.
